# Supplementary material for: The interactome of intact mitochondria by cross-linking mass spectrometry provides evidence for coexisting respiratory supercomplexes
Source: Mol Cell Proteomics. 2017 Dec 8;17(2):216–32. doi: 10.1074/mcp.RA117.000470 (PMC5795388; doi:10.1074/mcp.RA117.000470)
Supplement: Supplemental Data [file supp_RA117.000470_133922_0_supp_23978_fzffwf.zip › spectra_annotation/mito_spectra_annotation/57-1-3-1-1-1.pdf]

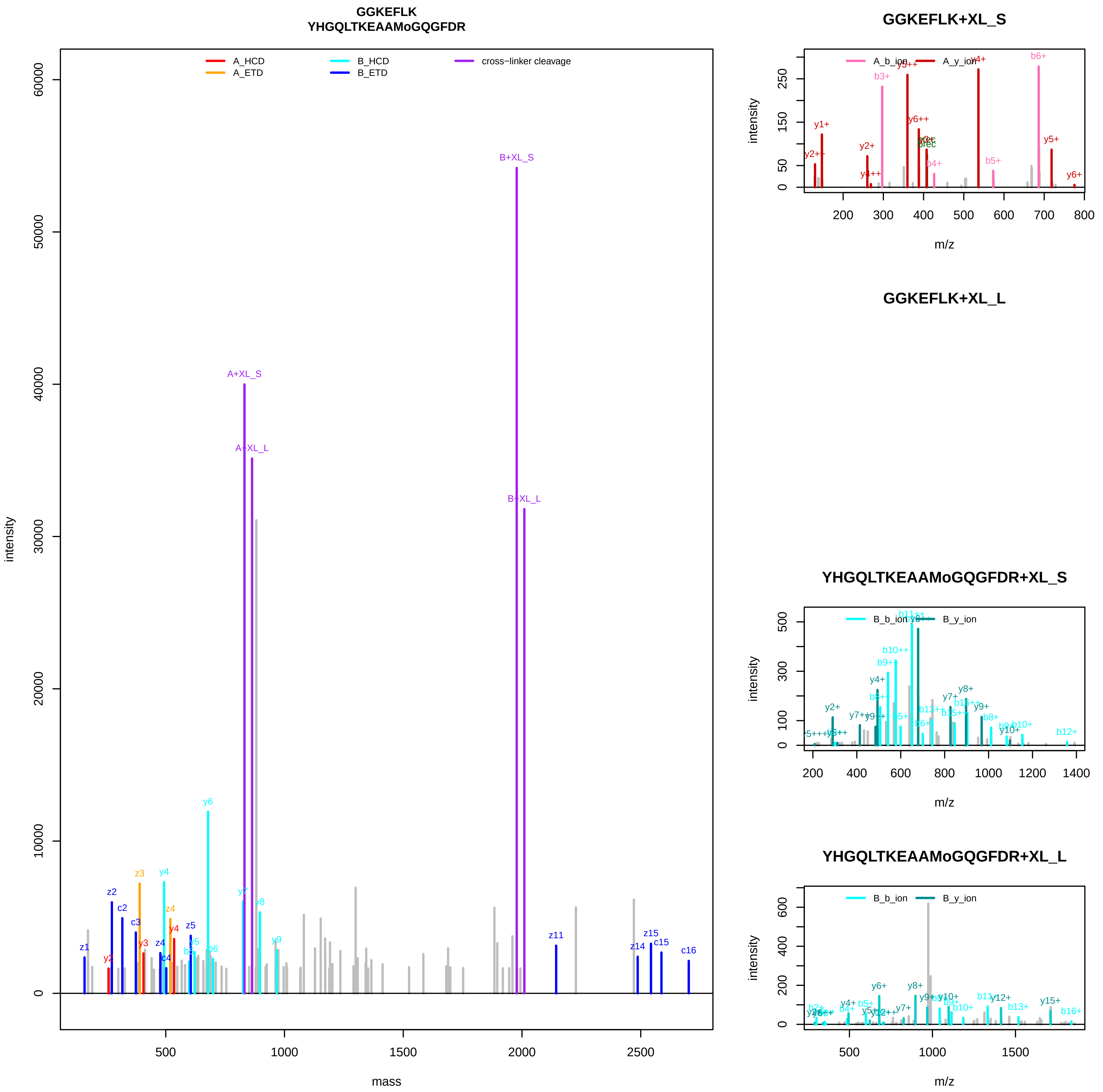
GGKEFLK
YHGQLTKEAAMoGQGFDR
A_HCD
A_ETD
B_HCD
B_ETD
cross−linker cleavage
intensity
mass
0
10000
20000
30000
40000
50000
60000
500
1000
1500
2000
2500
B+XL_S
A+XL_S
A+XL_L
B+XL_L
y6
z1
y2
z2
c2
c3
z3
y3
z4
y4
c4
z4
y4
z5
y5
b5
b6
y7
y8
y9
z11
z14
z15
c15
c16
GGKEFLK+XL_S
A_b_ion
A_y_ion
intensity
m/z
GGKEFLK+XL_L
YHGQLTKEAAMoGQGFDR+XL_S
B_b_ion
B_y_ion
intensity
m/z
YHGQLTKEAAMoGQGFDR+XL_L
B_b_ion
B_y_ion
intensity
m/z
